# Metformin for prevention of cesarean delivery and large-for-gestational-age newborns in non-diabetic obese pregnant women: a randomized clinical trial

**DOI:** 10.20945/2359-3997000000251

**Published:** 2020-06-05

**Authors:** Iramar Baptistella do Nascimento, Willian Barbosa Sales, Guilherme Dienstmann, Matheus Leite Ramos de Souza, Raquel Fleig, Jean Carl Silva

**Affiliations:** 1 Centro de Ciências da Saúde e do Esporte Universidade do Estado de Santa Catarina Florianópolis SC Brasil Centro de Ciências da Saúde e do Esporte (Cefid), Universidade do Estado de Santa Catarina (Udesc), Florianópolis, SC, Brasil; 2 Universidade da Região de Joinville Joinville SC Brasil Universidade da Região de Joinville (Univille), Joinville, SC, Brasil; 3 Centro de Educação do Planalto Norte Universidade do Estado de Santa Catarina Florianópolis SC Brasil Centro de Educação do Planalto Norte (Ceplan), Universidade do Estado de Santa Catarina (Udesc), Florianópolis, SC, Brasil; 4 Maternidade Darcy Vargas Universidade da Região de Joinville Joinville SC Brasil Maternidade Darcy Vargas; Universidade da Região de Joinville (Univille), Joinville, SC, Brasil

**Keywords:** Metformin, pregnancy, obesity, cesarean section, newborn

## Abstract

**Objective:**

To evaluate the use of metformin for preventing cesarean deliveries and large-for-gestational-age (LGA) newborn (NB) outcomes in non-diabetic obese pregnant women.

**Subjects and methods:**

This is a randomized clinical trial with obese pregnant women, divided into 2 groups: metformin group and control group, with followed-up prenatal routine. The gestational age of participants was less than or equal to 20 weeks and were monitored throughout entire prenatal period. For outcomes of delivery and LGA newborns, absolute risk reduction (ARR) and the number needed to treat (NNT) were calculated with a 95% confidence interval (CI).

**Results:**

357 pregnant women were evaluated. From the metformin group (n = 171), 68 (39.8%) subjects underwent cesarean delivery, and 117 (62.9%) subjects from the control group (n = 186) had intercurrence (p < 0.01). As for the mothers’ general characteristics, there was significance for marital status (p < 0.01). Maternal-fetal results presented reduced preeclampsia (p < 0,01). Primary prophylactic results presented an ARR of 23.1 times (95% CI: 13.0-33.4) with NNT of 4 (95% CI: 3.0-7.7) and no significant values for LGA NB (p > 0.01). Secondary prophylactic outcomes presented decreased odds ratio for preeclampsia (OR = 0.17, 95% CI: 0.10-0.41).

**Conclusion:**

The use of metformin reduced cesarean section rates, resulted in a small number of patients to be treated, but it did not reduce LGA NB. Administering a lower dosage of metformin from the early stages to the end of treatment may yield significant results with fewer side effects. Arch Endocrinol Metab. 2020;64(3):290-7

## INTRODUCTION

During pregnancy, changes and inherent risks associated to overweight and gestation are a concern, increasing adverse pregnancy outcomes especially when patients are obese or overweight from the beginning of the first trimester ( 1 ). The World Health Organization (WHO) uses an association between body mass index (BMI) and health risks as a classification. In this context, BMI values ranging from 25 to 29.9 kg/m^2^ indicate overweight, and results equal to, or greater than 30 kg/m^2^, are categorized as obesity ( 2 ).

In Brazil, obesity is present in 25 to 30% of pregnant women According to scientific literature (3), the eating behavior of pregnant women is one of the most prevalent causes for this. Thus, gestational obesity has become a problem that demands attention in this country ( 3 , 4 ) due to variables related to multifactoriality, methodological deficiencies, lack of control and efficient means to conduct weight gain (WG) during the gestation.

Recent research on overweight pregnant women has presented significant results for large-for-gestational-age (LGA) newborns (NBs) and for macrosomic infants ( 1 , 5 , 6 ). Studies have also pointed to a great association between increased outcomes of cesarean deliveries and mothers affected by obesity ( 7 , 8 ).

The impact of obesity in pregnant women represents a risk factor for women’s health, not only because of their excessive weight gain and its imminent intercurrences, but also because of innumerable problems that can be predicted in fetuses and newborns according to composite neonatal morbidities (CNMs), such as neonatal sepsis ( 9 ). Current decade research recognized the possible association between pre-gestational overweight and maternal-fetal intercurrences, identifying a relation between mother’s weight during pregnancy and NB fat mass (FM) ( 10 ).

Pharmacokinetic and pharmacodynamic metformin action reduce gluconeogenesis in the liver, which favors gestational outcomes ( 11 ). Furthermore, this drug is being used in the treatment of Polycystic Ovarian Syndrome (PCOS) and has an adjunct effect in patients submitted to chemotherapy for cancer treatment ( 12 , 13 ).

It is worth mentioning that acetylsalicylic acid (ASA) has been used with other treatments. Aspirin inhibits the synthesis of prostaglandins ( *PGS* ) through irreversible acetylation of fatty acids, which inactivates the cyclooxygenase ( *COX* ) enzyme ( 14 ). Thus, the use of ASA suggests prophylactic effects on maternal and perinatal outcomes, according to scientific literature on the prevention of pregnancy-specific hypertensive diseases (PSHD) and neonatal intercurrences ( 15 ).

Therefore, since metformin does not correlate with perinatal complications, and pregnant women preferred metformin to insulin treatment ( 16 ), we hypothesized that the use of this drug may result in a lower number of LGA NBs and cesarean deliveries (CDs) in pregnant women with a BMI ≥ 30. A consistent assumption is that in addition to hyperglycemia, obesity in pregnancy suggests a risk factor for LGA NBs and for higher cesarean rates in obese pregnant women who did not present a diagnosis of gestational diabetes mellitus (GDM) ( 5 ). In this way, this study aimed to evaluate the pharmacological action of metformin hydrochloride for the prevention of cesarean deliveries in obese pregnant women and for outcomes of LGA NBs.

## SUBJECTS AND METHODS

A randomized clinical trial was conducted from October 31, 2014 to December 31, 2017. This study is part of a larger research study that aimed to reduce the GDM in obese pregnant women using metformin. For the current study, 357 pregnant women with a diagnosis of obesity and adequate criteria to participate were selected. They were divided into two groups: a control group and a metformin hydrochloride intervention group. The treatment was developed at Darcy Vargas Maternity Hospital, (MDV) in the city of Joinville, Santa Catarina, which has a multidisciplinary service for obese pregnant women characterized as a risk group. Pregnant women are referred to the MDV outpatient clinic from the Basic Health Unit, and overweight and obese pregnant women only are seen and treated regularly on Thursdays.

Considering a scientific study outcome with a population of 298 pregnant women, 53 (17.8%) were categorized as obese, that is, with a BMI ≥ 30.0. Researchers identified an LGA NB rate of 15% in the perinatal outcomes of obese pregnant women. With the use of metformin, the present study considered a 46,6% reduction in the number of LGA NBs, from 15% to 7%, with a power of 90% and alpha error of 0.05. Thus, a sample of 165 pregnant women was obtained for each group ( 5 ).

The research was applied according to Resolution 466/12 regulations of the National Health Council. The confidentiality of pregnant women and their children was preserved. The study was approved according to Brazil Platform, CAAE (Certificate of Presentation for Ethical Appreciation) number: 34863514.1.0000.5366. The women were duly instructed, informed of research objectives and of their right to refuse participation at any time during this research, without prejudice or penalty of any nature to her or her baby. The integrity, safety and privacy of the information obtained during the study were maintained, and, concomitantly, the confirmation number corresponding to the RBEC (Brazilian Registry of Clinical Trials), U1111-1162-6908. This report follows recommendations of the Consolidated Standards Reporting Trial (CONSORT) ( 17 ).

The categorization of pregnant women’s weights occurred early in the morning, performed by the nursing team at the MDV triage, and subsequently, by current study researchers, according to WHO criteria for obese pregnant women (BMI ≥ 30 kg/m^2^). Patients were invited to attend lectures on gestational obesity and on the efficacy of metformin, its risks and benefits. The women who agreed to be part of the study signed two free and informed consent forms (ICF).

The study included all pregnant women without any type of diabetes, and those with a diagnosis of obesity, as per the World Health Organization criteria; a BMI ≥ 30 kg/m^2^; age 18 years or older; a single gestation; a primary cesarean section or obese pregnant women who had already performed the procedure in previous pregnancies; a gestational age (GA) less than 20 weeks of; and no pathologies interfering with delivery route and newborn. In addition, obese pregnant women should present following characteristics to be included: have no history or presence of pathologies related to liver, kidney, stomach or intestine; have no major drug allergy and/or other characteristic impairing drug absorption, distribution, excretion or metabolism. Subjects were excluded due to follow-up loss, that is, no adherence to treatment, research abandonment, or drug intolerance.

Follow-up was performed during the prenatal period, according to the basic routine recommended by National Health Department. All pregnant women were given standard prenatal care, receiving care from nutrition, nursing, physiotherapy and obstetrics services. MDV’s dietary guidance includes a small reduction in caloric intake of 24 kcal/kg/day ( 18 ). Along with standard treatment, pregnant women in the metformin group received 1,000 milligrams daily (mg/d) metformin: 500 mg at breakfast and at dinner. The control group received standardized hospital treatment only. The intention of using a lower dosage, compared to previous studies, was to observe the results of the study showing the lowest levels of intolerance to metformin in pregnant women. However, this was not the main objective of the study.

During the first visit to the gestational obesity outpatient clinic, the participants were randomized by a computerized algorithm, using Microsoft Excel (Microsoft, Redmond, WA, USA), which generated a random allocation order list in a non-fixed proportion, divided into two groups: a study group, which was treated with metformin and received guidance on diet and physical activity; and a control group, which received guidance on diet and physical activity only. Participants received a coded seal on their prenatal follow-up records, which identified them as participants of the multidisciplinary research team during outpatient visits throughout the study. All patients were identified through a specific research form containing: participant’s name; date of birth; age; marital status; occupation; educational level; ethnicity; BMI; gestational age (GA) at entry; allergy to metformin; number of pregnancies; interval between deliveries; age at birth of first child; type of delivery; abortions; use of medication during pregnancy; renal, or gastrointestinal disease; diagnosis of liver; GDM diagnosis.

The data collected for maternal characteristics were: maternal age; ethnicity; marital status; schooling; number of pregnancies; GA of entry; BMI in all three trimesters of gestation: upon arrival (less than 20 weeks), second trimester (24-28 weeks) and third trimester (33-35 weeks). Consequently, for maternal-fetal outcomes, the following information was observed: gestational diabetes mellitus (GDM) acquired; preeclampsia (PE); prematurity; SGA NB; Apgar scores in the 1st and 5th minutes; and admittance to neonatal intensive care unit (NICU). It is noteworthy to mention the relation between gestational obesity and increased numbers of cesarean births and LGA NBs. Further, these outcomes compromise both mother and NB ( 1 , 5 ). In this manner, the primary objective of this study was to identify the number of cesarean births and LGA NBs to non-diabetic obese pregnant women with the use of the drug. All other outcomes evaluated, including that of DGM, were for secondary analysis. In the present article, the main outcomes evaluated were: Absolute risk reduction (ARR) and number needed to treat (NNT) for CD and LGA NBs. The Lubchenco curve ( 19 ) was used for this study. Widely used in Latin American maternity hospitals, the Lubchenco curve classifies children below the 10^th^ percentile as small for gestational age (SGA); children between the 10^th^ and 90^th^ percentiles as appropriate for gestational age (AGA); and children above the 90^th^ percentile as LGA NBs ( 19 ). Variables were statistically treated using the Statistical Package for Social Sciences (SPSS), version 21.0. At first, all data were analyzed descriptively. For continuous variables, analysis was performed through the calculation of means and standard deviations. Student’s t-test was used for hypothesis analysis and comparison of means between groups, and the non-parametric Mann-Whitney test was used when normality was rejected. The Kolmogorov-Smirnov test was used as support to verify the normality of population. Chi-square or Fisher’s exact tests were applied for frequencies smaller than five to test homogeneity of groups in relation to proportions.

Risk factors were identified by univariate analysis, which compared variables categorized by Q^2^ test. Absolute risk reduction (ARR) and the number needed to treat (NNT) for CDs and LGA NBs were calculated. ARR is the result after intervention, that is, the prevalence of sample baseline risk that remains and will reduce after intervention has developed. The NNT is the number of patients who must be treated with a specific drug to avoid an outcome of disease. Relative Risk (RR) is the ratio between prevalent absolute risk and the result of absolute risk found after intervention ( 20 ). For NB outcomes, the multinomial logistic regression model was set up containing adjusted values for odds ratios. For all research results, a 95% confidence interval (CI) was adopted considering significant values when p < 0.05.

## RESULTS

**Figure 1:**
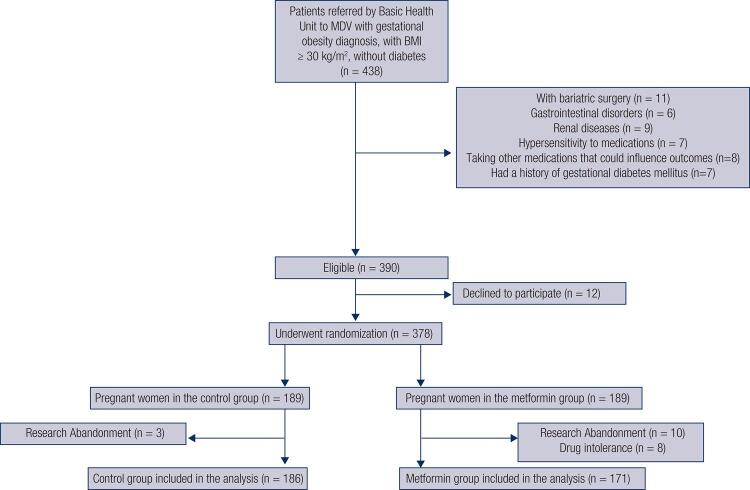
Participants flowchart at each stage of the study.

Source: Author (2018).

The basic health unit referred 438 obese pregnant women with a BMI ≥ 30 (kg/m^2^), classified as high-risk pregnancy for specialized care at the MDV obesity outpatient clinic. Of these, 48 pregnant women had characteristics that were not compatible with the study: 11 pregnant women had had bariatric surgery; 6 had previous experience with gastrointestinal disorders (dyspepsia, gastroenteritis, esophagitis and gastritis); 9 had kidney disease (history of pyelonephritis, renal cysts or renal failure); 7 already knew of their hypersensitivity to medication; 8 were taking other drugs that could influence outcomes; and 7 had a history of gestational diabetes mellitus. Although 390 women were eligible to participate in the study, 12 refused to be part of the research, resulting in 378 pregnant women for randomization. The pregnant women were divided into two groups: 189 in the control group and 189 in the metformin intervention group. Three women from the control group abandoned the study; as for the intervention group, 10 abandoned the research and 8 presented drug intolerance, indicating a 4.2% rate of pregnant women presenting side effects after drug use. The remaining 357 obese pregnant women were included in the analysis, were subject to all prenatal care and delivered their babies at MDV. Finally, we obtained 186 (52.1%) pregnant women for the control group and 171 (47.9%) for the metformin group, detailed in Figure 1 .

Table 1 presents the mothers’ baseline characteristics in a comparison between the two groups: control and metformin. Qualitative variables did not identify relevance for factors related to ethnicity and education (p > 0.05). However, marital status revealed significant values (p < 0.01). As for quantitative aspects, the gestational BMI and factors corresponding to maternal age, number of gestations and gestational age (GA) did not indicate statistical differences (p > 0.05).

**Table 1:** Patients baseline characteristics

	Total Sample (N = 357)	Study group		Control vs. Metformin P Value

		Control (N = 186)	Metformin (N = 171)	
Age (SD)	29.1 (6.2)	29.6 (6.1)	28.6 (6.2)	0.12*
Ethnicity				
White	279 (78.2)	140 (75.3)	139 (81.3)	0.38**
Black	47 (13.2)	28 (15.1)	19 (11.1)	
Other	31 (8.7)	18 (9.7)	13 (7.6)	
Marital status				
Single	58 (16.2)	26 (14.0)	32 (18.7)	< 0.01**
Married	259 (72.5)	147 (79.0)	112 (65.5)	
Other	40 (11.2)	13 (7.0)	27 (15.8)	
Education				
< 18 years	50 (14.0)	24 (12.9)	26 (15.2)	0.23**
Middle school	105 (29.4)	50 (26.9)	55 (32.2)	
High school	166 (46.5)	88 (47.3)	78 (45.6)	
College	36 (10.1)	24 (12.9)	12 (7.0)	
Number of pregnancies (SD)	2.7 (2.0)	2.7 (2.0)	2.6 (2.0)	0.73*
Intake GA (SD)	11.4 (3.2)	11.3 (3.1)	11.5 (3.2)	0.62*
Gestational BMI (≥ 30 kg/m^2^) (SD)				
Intake BMI	37.4 (5.2)	37.2 (5.7)	37.5 (4.6)	0.26***
3^rd^ Trimester BMI	38.8 (6.2)	38.9 (6.3)	38.7 (6.1)	0.33***

SD: standard deviation; GA: gestational age; BMI: body mass index. Statistical tests: *T test; **Chi-square. ***Mann-Whitney.

Table 2 shows a risk ratio comparison for the use of metformin to prevent CD and LGA NB, in the respective groups. In the study group (n = 171), where metformin was administered to mothers, the incidence rate of cesarean deliveries was 39.8%. In the control group (n = 186), CDs occurred for 62.9% of pregnant women, presenting significant values (p < 0.01). ARR was equal to 23.1 (95% CI: 13.0- 33.24), with an NNT of 4 (95% CI: 3.0 -7.7). In LGA NB prevention, the intervention with metformin group (n = 171) had 30.4% of LGA NBs, and the control group (n = 186) 27.4%. Thus, parameters did not present significant values (p > 0.05).

**Table 2:** Metformin in the prevention of cesarean births and large for gestational age (LGA) newborns (NB)

Outcomes	Control Group (N = 186) N (%)	Metformin Group (N = 171) N (%)	ARR (CI 95%)	NNT	P
Cesarean Sections	117 (62.9)	68 (39.8)	23.1 (13.0-33.24)	4 (3.0-7.7)	< 0.01
LGA NBs	51 (27.4)	52 (30.4)	-	-	0.53^†^

ARR: absolute risk reduction; N: number; NB: newborn; LGA: large-for-gestational-age; CI: confidence interval; NNT: number needed to treat. Statistical tests: ^†^ Chi-square test.

Consequently, the following maternal-fetal outcomes were assessed: GDM, PE, prematurity, newborn weight, SGA, Apgar 1st. and 5th minutes, NICU. Among the results evaluated with metformin use, only PE presented chances of reduction in the incidence (OR = 0.17, 95% CI 0.10-0.41) (p < 0.01), according to Table 3 .

**Table 3:** Maternal-fetal complications, according to study group

Outcomes	Group	N (%)	Adjusted OR (CI 95%)	P Value
GDM	Control	15 (8.1)	-	0.34
	Metformin	10 (5.8)	0.66 (0.28-1.55)	
Preeclampsia	Control	33 (17.7)	-	< 0.01
	Metformin	6 (3.5)	0.17 (0.10-0.41)	
Prematurity	Control	9 (4.8)	-	0.35
	Metformin	9 (5.3)	1.68 (0.55-5.14)	
SGA NB	Control	3 (1.6)	-	...
	Metformin	0 (0.0)	-	
Low Apgar-1	Control	13 (7.0)	-	0.39
	Metformin	14 (8.2)	1.43 (0.63-3.24)	
Low Apgar-5	Control	1 (0.5)	-	...
	Metformin	0 (0.0)	-	
NICU	Control	3 (1.6)	-	0.52
	Metformin	3 (1.8)	1.89 (0.27-13.17)	

GDM: gestational diabetes mellitus; NB: newborn; SGA: small-for-gestational-age; NICU: neonatal intensive care unit. Adjusted Variables: age, ethnicity, marital status, schooling, number of pregnancies, gestational age, pre-gestational body mass index, development of GDM, development of preeclampsia, preterm newborns, 1st and 5th low minutes (< 7) and NICU admission.

## DISCUSSION

The present study showed the impact of metformin on obese pregnant women to prevent cesarean delivery and perinatal outcomes. It was possible to evaluate metformin use in the incidence of cesarean deliveries and weight of newborns and to quantify the ARR and NNT for the main objectives of the study. Further, this study pointed to an ARR in the incidence of cesarean deliveries and not in LGA NB outcomes.

Regarding general characteristics of the mothers, the only significance was marital status (p < 0.01). It should be noted that the study presented a 51.8% rate of cesarean deliveries, which, according to bibliographical findings, confirms the strong relation between the BMI outside appropriate standards and CDs and LGA NBs ( 5 ). Likewise, statistical percentages increase when these outcomes are related to married pregnant women above appropriate weight and PSHD ( 5 , 21 ).

Metformin has been used in obese pregnant women with GDM, yet the relationship between pharmacokinetic and pharmacodynamic mechanisms is not fully understood. However, metformin activates the enzyme protein kinase, involved in controlling body energy and metabolic substrate, reducing gluconeogenesis ( 22 , 23 ). Other investigators have found that metformin may favor a better redistribution of peripheral and visceral fat in, although they did not observe differences in body fat percentage ( 24 ).

In literature, researchers used different GA to initiate metformin therapy. A recent study began treatment at 12 to 18 weeks of gestation, reducing WG in obese pregnant mothers ( 25 ). Other authors have instituted use of the drug beginning in the first trimester ( 26 ). Our average for metformin use was established from 11 to 16 weeks of pregnancy, which, in line with the bibliography, ratified the relevance of starting administration before 20 weeks of pregnancy.

The metformin dosage to be administered in pregnancy has become relevant in the design of results. A current meta-analysis with randomized clinical trials concluded that the drug may reduce risks in the outcomes of overweight pregnant women ( 27 ). Research using metformin from first trimester at a dosage of 1,700 mg/d concluded that complications during pregnancy and postpartum decreased in women with PCOS ( 12 ). Consecutively, researchers identified significant differences in maternal outcomes with 500 to 3000 mg ( 25 ), while another study had significant differences with 500 to 2,500 mg/d ( 28 ). The present study used 1,000 mg/d in the prevention of cesarean deliveries and LGA NB outcomes. This dose of 1,000 mg is different from dosages used in other studies.

Regarding the perspective of pregnant women, a previous study demonstrated a better acceptance of metformin administration, when compared to insulin (76.6% *vs.* 27.2%), respectively (p < 0.01) ( 16 ). An impact study, with an initial dose of 500 mg/d (once or twice a day) gradually increased over a period of two to three weeks to reach a dosage of 2,500 mg/d, showed a 4.2% intolerance rate to the drug, with the metformin plus insulin use ( 16 ). The Vanky et al studies showed the intolerance rate to this drug was 16.7% ( 12 ) among obese pregnant women using 2,000 mg/d. Other studies that used up to 3,000 mg/d noted that 41.8% of women stopped taking their pills due to gastrointestinal side effects, and another 40% had to reduce their dose during treatment ( 25 ). In the current study, only 4.2% of the 189 pregnant women who used the drug presented intolerance to the pre-established dosage of 1000mg/d. Regarding the side effects of metformin, vomiting, nausea and diarrhea are most frequent ( 29 ).

A similar study with obese pregnant women who used the drug did not reduce chances of cesarean delivery (OR = 0.93, 95% CI 0.62-1.38) (p = 0.79) ( 25 ). A current meta-analysis using two clinical trials with obese pregnant women without DMG was not consistent with our results on metformin in cesarean delivery outcomes. No significant values were found for risk reduction for cesarean section (RR 0.91, 95% CI 0.76, 1.09) ( 30 ). A recent clinical trial with obese pregnant women regarding prevention of primary cesarean sections resulted in 46/222 (21%) in the placebo group versus 42/219 (19%) in the metformin group (OR 0.90, 95% CI 0.57, 1.45) ( 30 ). This present study indicated an RRA of cesarean delivery in the metformin group of 23.1 (95% CI 13.0-33.24), as it was necessary to treat 4 pregnant women to prevent CD outcomes (NNT = 4), (p < 0.01).

Nevertheless, there was no RRA to prevent LGA NBs. That is, no difference was found between the two groups (p > 0.05). This is in agreement with an already published scientific finding, which, even after drug use, did not show a reduction in odds risk for LGA NBs (OR = 1.11, 95% CI 0.65-1.90), (p = 0.79) (p > 0.05) ( 25 ).

Other researchers have found metformin has no effect on newborn weight percentile in obese pregnant women ( 28 ). An impact study showed greater measures of subcutaneous fat in children exposed to metformin, but total body fat was similar in children whose mothers administered insulin during pregnancy ( 31 ).

Infant follow-up is necessary to better evaluate the use of metformin and to analyze the impact of the medication on intrauterine exposure. Nonetheless, when metformin was used during pregnancy, no metabolic syndromes throughout the children´s lives have manifested in other studies ( 32 ). A study concluded that the drug was not associated with perinatal complications when compared to insulin ( 16 , 33 ) in women with GDM who used only metformin during gestation as a preventive alternative.

As for ASA, the incidence of LGA NBs of pregnant mothers treated with aspirin was of concern in scientific literature, resulting in 40.2% *vs.* 26.6% in the placebo group, (P = 0.005) ( 34 ). Regarding other secondary findings, this study identified only reduced odds for PE with lower percentages of 3.5% for metformin group vs. 17.7% for control group, indicating lower chances for the intervention group (OR = 0.17, 95% CI = 0.10-0.41). This result is analogous to results of other authors (OR = 0.24, 95% CI = 0.10-0.61) ( 24 ). It is worth mentioning that a review study with 59 clinical trials showed, although with limited statistical values, lower risks for PE incidence, 17% (RR = 0.83, 95% CI = 0.77-0.89), with an NNT of 72 pregnant women receiving aspirin ( 35 ). Thus, PE reduction in this research seems to have favored decreased cesarean values in metformin group. That considered, a strong relation between cesarean delivery and PE was reported in a recent study ( 36 ).

As for the limitations, we can affirm it was difficult to guarantee generalization capacity, which favors external validation. Nonetheless, the present study preserved internal validity, as the selected patients fit the conditions of a specific group of non-diabetic pregnant women and were categorized as obese and matched inclusion criteria previously established in the protocol. The acquisition of the sample was another positive point, as it was compatible with predicted statistical values, benefitting the quality of results.

In conclusion, the use of metformin showed a reduction in cesarean section rates with a small number of patients to be treated, but it did not reduce LGA NB rates. A lower dosage administered from the early stages to the end of treatment may yield significant results with fewer side effects.
